# Relationship between Immune Cells, Depression, Stress, and Psoriasis: Could the Use of Natural Products Be Helpful?

**DOI:** 10.3390/molecules27061953

**Published:** 2022-03-17

**Authors:** Alessio Alesci, Eugenia Rita Lauriano, Angelo Fumia, Natasha Irrera, Enza Mastrantonio, Mario Vaccaro, Sebastiano Gangemi, Antonello Santini, Nicola Cicero, Simona Pergolizzi

**Affiliations:** 1 Department of Chemical, Biological, Pharmaceutical and Environmental Sciences, University of Messina, Viale Stagno d’Alcontres, 31, 98166 Messina, Italy; elauriano@unime.it (E.R.L.); spergolizzi@unime.it (S.P.); 2 Department of Clinical and Experimental Medicine, University of Messina, Viale Gazzi, 98147 Messina, Italy; angelofumia@gmail.com (A.F.); sebastiano.gangemi@unime.it (S.G.); 3 Department of Clinical and Experimental Medicine—Section of Pharmacology, University of Messina, 98125 Messina, Italy; natasha.irrera@unime.it; 4 Azienda Sanitaria Provinciale Messina, 98124 Messina, Italy; mastrenz.a@hotmail.it; 5 Department of Clinical and Experimental Medicine—Section of Dermatology, University of Messina, 98125 Messina, Italy; mario.vaccaro@unime.it; 6 Department of Pharmacy, University of Napoli Federico II, 80131 Napoli, Italy; 7 Department of Biomedical and Dental Science and Morphofunctional Imaging, University of Messina, 98125 Messina, Italy

**Keywords:** psoriasis, immune system, immune cells, depression, stress, natural products

## Abstract

Psoriasis is one of the most widespread chronic inflammatory skin diseases, affecting about 2%–3% of the worldwide adult population. The pathogenesis of this disease is quite complex, but an interaction between genetic and environmental factors has been recognized with an essential modulation of inflammatory and immune responses in affected patients. Psoriatic plaques generally represent the clinical psoriatic feature resulting from an abnormal proliferation and differentiation of keratinocytes, which cause dermal hyperplasia, skin infiltration of immune cells, and increased capillarity. Some scientific pieces of evidence have reported that psychological stress may play a key role in psoriasis, and the disease itself may cause stress conditions in patients, thus reproducing a vicious cycle. The present review aims at examining immune cell involvement in psoriasis and the relationship of depression and stress in its pathogenesis and development. In addition, this review contains a focus on the possible use of natural products, thus pointing out their mechanism of action in order to counteract clinical and psychological symptoms.

## 1. Introduction

**Figure 1:**
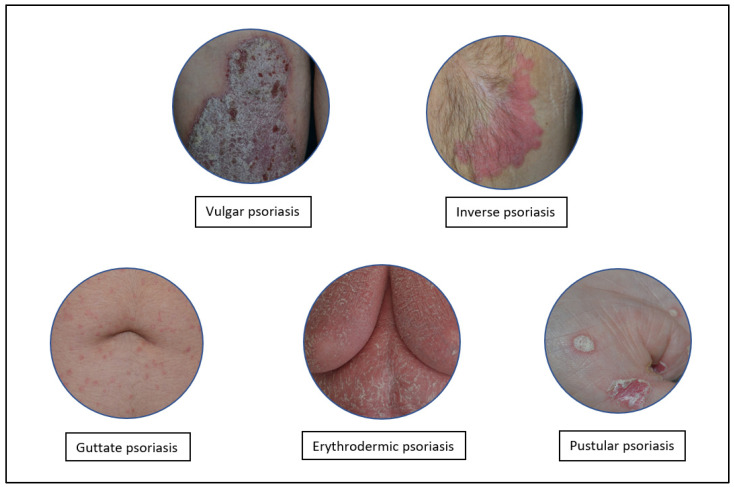
Different types of psoriasis.

Psoriasis is one of the most common chronic inflammatory skin diseases that affects about ~2%–3% of the worldwide adult population [1,2]. Psoriasis affects both males and females, without prevalence of gender [3], and its clinical lesions start to appear between 20 and 30 years of age, although children and adolescents may also be affected [4]. This pathology can be classified into several forms: *Psoriasis vulgaris*, *P. guttata*, *P. pustolosa*, *P. erythrodermica*, and *P. inversa* [5] (Figure 1).

The pathogenesis of the disease is quite complex, but an interaction between genetic and environmental factors has been recognized [6,7,8]. Psoriasis is characterized by skin lesions that appear as erythematous and reddened plaques with silvery lamelliform scales, sometimes painful and itchy [9], especially in the elbows, knees, and scalp [10]. These plaques are often the result of an abnormal proliferation and differentiation of keratinocytes, thus resulting in dermal hyperplasia [11], immune cell infiltration, and increased capillarity [12]. Because of the significant role of immune cells in the pathogenesis of psoriasis, this disease is also classified as an autoimmune disease. In fact, different studies have already shown that the activation of T helper (Th)1 lymphocytes leads to an increase in interferon (IFN)-γ and tumor necrosis factor (TNF)-α levels [13]. CD4+ T cells also produce interleukin (IL)-17, which is responsible for the abnormal cell proliferation and stimulation of keratinocytes, thus leading to cytokine release [14,15]. In this context, the IL-23/IL-17 axis would play a crucial role in psoriatic lesion formation [16,17]. These cytokines, with particular reference to IL-17, are also produced by other immune cells, such as γδ T cells, natural killer (NK) cells, NKT cells, and innate lymphoid cells (ILCs) [18], which are involved in the pathogenesis of psoriasis [19]. Psoriasis also affects the psychological plane, reducing the quality of life of patients [20,21]: the appearance of skin lesions can cause discomfort in patients and may increase layoff and unemployment rates and depressive states up to suicide rates [22,23]. Not only the affected patients are stressed out, but also stressful conditions may represent contributing causes of psoriasis [24]. In fact, stress can activate an inadequate response of the hypothalamic–pituitary–adrenal (HPA) axis, thus stimulating corticotropin-releasing hormone (CRH) and vasopressin release in the hypothalamus with the consequent adrenocorticotropic hormone (ACTH) increase [25]. ACTH regulates glucocorticoid secretion [25], but psoriatic patients are extremely reactive to stress and show reduced cortisol levels, resulting in immune system hyperactivity and increased inflammation [25,26,27]. As of today, the therapeutic approaches used for the treatment of psoriasis mainly act by reducing its severity or even suppressing symptoms, but no therapy is currently 100% effective. The most used treatments are monoclonal antibodies against TNF-α, IL-17, and IL-23 and topical cortisone-based ointments that contribute to the disappearance of plaques [28,29]. However, as mentioned, these approaches are not definitively curative and are long-term therapies, thus causing a weakening of the immune system [30]. For this reason, a growing interest is aimed at alternative treatments based on natural product use, such as some nutraceuticals and polyphenols whose mechanism of action is mainly antioxidant and that are recognized to be safe [31,32,33,34,35,36].

In this review, immune cell involvement in psoriasis pathogenesis and the psychological consequences of psoriatic patients are reported. In addition, an overview of some natural products that might be used to manage this complex disease is designed.

## 2. Cell Types Involved in the Pathogenesis of Psoriasis

### 2.1. Keratinocytes

**Figure 2:**
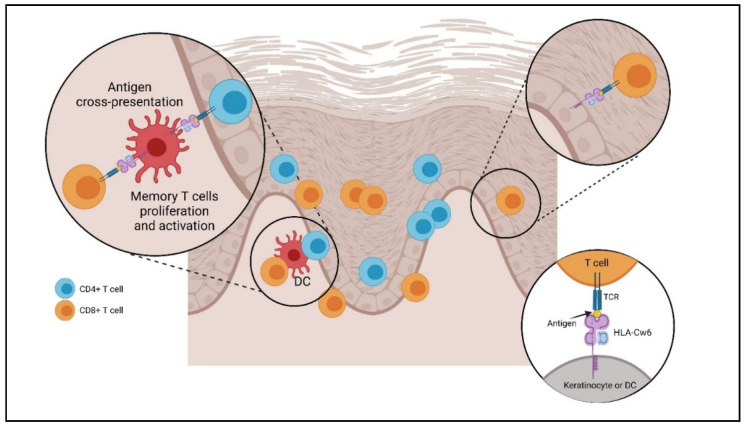
Role of HLA-Cw6 in psoriasis pathogenesis. Created with BioRender.com (accessed on 18 February 2022).

Keratinocytes are the most distributed cells in the skin characterized by a turnover that occurs in about 50 days under physiological conditions and in 5 days only during psoriasis, demonstrating an increased cell turnover and in particular of keratinocytes [37,38,39]. These cells play a key role in maintaining the inflammatory balance [40]: any mechanism that can alter this balance can lead to the development and evolution of chronic skin diseases, including psoriasis [41]. Activated keratinocytes may contribute to the release of chemokines, which are able to attract different defense cells to the site of phlogosis [42]. In particular, keratinocytes regulate the expression of the antimicrobial peptide cathelicidin LL-37 [43] and produce chemokine ligand (CXCL)8, also known as keratinocyte-derived chemokine 8 (KC8), which is responsible for neutrophil infiltration [44]; CXCL10; and chemokine receptor (CXCR) 3, which activate monocytes and Th1 cells [45]. Furthermore, chemokine ligand (CCL) 20 and IL-18 are implicated in the enrolment of Langerhans cells (LCs), dendritic cells (DCs), and cutaneous lymphocyte-associated antigen (CLA) T cells [46,47]. In addition, keratinocytes play the role of antigen-presenting cells, expressing the human leucocyte antigen (HLA) or major histocompatibility complex (MHC) [48]. HLA-Cw6 is a valid candidate for functional involvement in psoriasis. HLA-Cw6 participates in the cross-presentation of the antigen on a DC surface, resulting in the activation of CD8+ antigen-specific T cells. Cross-priming depends on the help of CD4+ T cells in the presentation of intracellular antigens in the dermis (activation of resident memory T cells) and lymph nodes (activation of naive T cells). Activated CD8+ T cells can migrate into the epidermis by binding to HLA-Cw6 keratinocytes, triggering the release of cytokines, chemokines, and proinflammatory mediators. This process increases inflammation and induces the proliferation of keratinocytes. The cyclical mechanism triggered in this way causes the psoriatic injury (Figure 2) [49,50]. Among the receptors expressed by keratinocytes and immune cells, the most important group is represented by the Toll-like receptors (TLRs), a class of phylogenetically conserved proteins [51,52,53,54], both in physiological and pathological conditions [55]. A reduced and increased expression was found in keratinocytes obtained from psoriatic plaques of TLR-5 and TLR-1, respectively [56,57]. Furthermore, keratinocytes’ structural integrity is maintained by keratins (KRTs), which play an important role in the pathogenesis of psoriasis [58]. In stress conditions, keratinocytes release damage-associated molecular patterns (DAMPs), which activate different molecular pathways, including the mitogen-activated protein kinase pathway (MAPK) and the transcription factors nuclear factor kappa-light-chain-enhancer of activated B cells (NF-kB) and nuclear factor erythroid 2-related factor 2 (NRF-2), which in turn mediate KRT6, KRT16, and KRT17 transcription. A high proliferative stage under pathological conditions is indicated by the increased expression of KRT16/17–KRT6; in particular, KRT17 expression is negligible in normal epidermis but is overexpressed in hyperproliferative diseases, such as psoriasis. KRT17 activates cell proliferation through the mTOR–AKT signaling, an important intracellular pathway involved in the regulation of the cell cycle. KRT17 may also act as an autoantigen, thus activating DCs and triggering the cytokine cascade, which in turn acts on keratinocytes by activating extracellular signal-regulated kinases (ERK)1/2 and signal transducers and activators of transcription (STAT)1/3. This activation further increases keratin gene expression, creating a loop that contributes to psoriasis appearance [59]. All these reported pieces of evidence highlight the important role played by keratinocytes in the formation and development of this chronic inflammatory disease.

### 2.2. Dendritic Cells

Different types of dendritic cells can be detected in the skin under both physiological and pathological conditions [60,61]. These cells can act as antigen-presenting cells (APCs), but also as an important source of chemical mediators of inflammation, such as TNF-α and IL-23 [62].

#### 2.2.1. Plasmacytoid Dendritic Cells (pDCs)

Plasmacytoid dendritic cells (pDCs) are a cell population present in the blood and secondary lymphoid organs [63]. They are involved in the antiviral immune response and can secrete large amounts of IFN-α [64]. Once stimulated by the viral antigen, these cells can differentiate into DCs [65] or dendritic myeloid cells (mDCs), which can modulate the inflammatory response by Th1 lymphocytes [66]. pDCs may accumulate in peripheral tissues during noninfectious phlogosis states, such as psoriasis [67]. pDCs have also been found in inflamed tissues of psoriasis-like diseases, such as systemic lupus erythematosus [68] and *rheumatoid arthritis* [69]. The activation of pDCs occurs by TLR-7 and TLR-9 [70,71]. This activation would therefore represent the trigger point of the pathogenesis of psoriasis, leading to a mechanism that contributes to the appearance of the psoriatic phenotype. Several studies, including murine models, have shown that an increase in IFN-α produced by pDCs can be found in the skin damaged with psoriatic plaques [67,72]. In addition to viral antigens, their recruitment and activation can also be induced by keratinocyte-released chemokines, such as CXCR4, CXCR3, and CCR5 [73,74]. The monoclonal antibody antiblood DC antigen 2 (BDCA2) is able to inhibit the development of psoriatic lesions, blocking the activation of pDCs [72]. Further studies using imiquimod [75], a synthetic imidazoquinolinone recognized by TLR-7 [76], have demonstrated the induction and activation of pDCs with IFN-α release [77].

#### 2.2.2. Myeloid Dendritic Cells (mDCs)

Myeloid dendritic cells (mDCs) are highly represented in psoriatic skin lesions [62] and may be recognized as CD11c + CD1c (phenotypically immature cells) and CD11c + DC-LAMP + DEC-205/CD205 + BDCA-1+ (phenotypically mature cells). CD11c + CD1c cells are responsible for the production and release of TNF-α, IL-6, IL-20, IL-23, and IL-12 and produce inducible nitric oxide synthase (iNOS) [78]. Thus, these cells are particularly involved in the formation of psoriatic lesions [79,80,81,82], acting as mediators of inflammation and playing a key role in the pathogenesis of this chronic disease [60]. Although these cells are capable of releasing a large number of chemical mediators of inflammation, they primarily secrete IL-23, which interacts with the IL-17-mediated immune response [80,82]. Several studies have also shown that the presence of mDCs in psoriatic skin is 30 times higher than in healthy skin [78]. The CD11 + Dc-LAMP + DEC-205/CD205 + BDCA-1+ mDCs may be considered APCs, and their number does not appear to increase in psoriasis-injured skin compared with that in healthy skin [81]. The mDCs can also be activated and recruited by keratinocytes releasing CCL20 and promoting a recall of the Th17 lymphocytes to the phlogosis site [12,83]. The data collected clearly show not only the involvement of mDCs in the pathogenesis of psoriasis but also the crucial role that these cells play in the development of the disease.

#### 2.2.3. Langerhans Cells (LCs)

Langerhans cells (LCs) are specialized dendritic cells with a defense role through antigen recognition and phagocytosis and are characterized by the presence of Birbeck granules in their cytoplasm, which are involved in endocytosis processes [84]. Studies on murine models have shown that LCs can play a role in acute psoriasis [85], increasing in number, and then decreasing after administration of an anti-IL-β antibody [86], which is responsible for the migratory deficiency and inhibition of LCs [87]. LCs appear to be involved in perpetuating psoriatic inflammation. LCs release different inflammatory chemokines [88], including IL-23 [89,90]. Further studies have shown that DCs can secrete TNF-α and iNOS [79,82] and can activate allogeneic T cells and induce the release of IL-17, IL-22, and IFN-γ [17], such as LCs in psoriatic skin [88,90]. Other studies have described a protective and a modulating role in situ played by LCs through IL-10 release [91] and neutrophil recruitment [92], respectively. LCs’ role is not fully understood, but these cells appear to be involved in perpetuating inflammation and psoriatic injury.

### 2.3. Innate Lymphoid Cells (ILCs)

Innate lymphoid cells (ILCs) are heterogeneous immune cells involved in the formation of lymph nodes during fetal life and the remodeling of organs and tissues [93]. ILC2s produce type 2 cytokines (e.g., IL-4, IL-5, IL-9, IL-13) that are implicated in the immune response to allergens, helminths, cancer, and viruses [94]. ILC2s have also been identified in greater levels in tissues with allergy symptoms, such as nasal polyps in patients with chronic rhinosinusitis and the skin of individuals with atopic dermatitis [95]. Elevated concentrations of ILC2s in the blood are linked to atopic dermatitis, while high concentrations of ILC3 are linked to psoriasis [96]. ILC3 releases IL-17 and IL-22 and expresses NKp44, a natural cytotoxic receptor (NKR) involved in viral antigen recognition and interactions with other immune cells [97,98,99]. The presence of these cells in psoriasis-injured skin is higher than in noninjured skin [98].

#### Natural Killer Cells (NKs) 

Natural killer cells (NKs) belong to the ILC family. They maintain the state of health of the body and show cytotoxic and effector activity [97]. The cytotoxic effect is explained by the release of substances that induce cell lysis, such as granzyme, perforin, and granulysin (GNLY) [19], up to apoptosis induction [100,101]. NKs are found in excess in psoriatic lesions [102]; NKs represent about 8% of the entire cell population infiltrated into the lesion and express cytokines involved in psoriatic processes, such as IL-2, TNF-α, and IFN-γ as well as IL-17 and IL-22 [103,104]. Furthermore, they stimulate chemokine release by keratinocytes, useful for the in situ recall of other immune cells, implementing the inflammatory state [105]. NKs can express membrane NK receptors, such as killer immunoglobulin-like receptor (KIR) [106]. HLA-G is a ligand of KIR-2DL4 [107].

### 2.4. T Cells (Lymphocyte T Helper, T Cytotoxin γδ T, Natural Killer T Cells)

T cells are responsible for innate immunity, acting as sentinels and intervening in counteracting pathogenic antigens. They express TCRs (T cell receptors) consisting of an α chain and a β chain; however, T cells γδ T express receptors consisting of a γ chain and a δ chain [108]. T lymphocytes are widely represented in psoriasis-induced skin lesions [109] and are responsible for the release of IFN-γ, TNF-α, and IL-17 [109]. They also act in concert with keratinocytes, inducing the expression of HLA and IL-6 [109,110]. Specifically, Th, CD4+, and cytotoxic T lymphocytes (CD8+) are involved in the development of the disease [17,111]. Studies in immunodeficient mice have shown that CD4+ is among the first promoters of psoriasis [112]. In addition, CD4+ cell activation causes in situ recall and CD8+ cell stimulation, which are also involved in the pathogenesis of psoriasis [113]. The early infiltration of cytotoxic T lymphocytes, and not of Th lymphocytes, induces the phlogistic state typical of psoriasis [114,115]. In addition, keratinocytes express HLA-C*0602, which has antigens typical of TCD8+ cells and not CD4+ cells [49]. The efficacy of treatment with cyclosporin A [116,117] correlated with T lymphocyte inhibition and IL-2 reduction [116] has highlighted the significant role played by these cells in the pathogenesis of the disease. CD4+ Th17 can release IL-17, IL-23 [118], IL-22 [119], IFN-γ, and TNF-α. Th22 cells together with NK cells contribute to IL-22 release [119]. Some studies have shown that the γδ T cells are the T cells most involved in the release of IL-17 [17,120] and express different chemokines, such as CCR6, thus implementing the inflammatory state [120]. Natural killer T cells (NKTs) are a subpopulation of T cells that secrete IL-4 and IFN-γ and also represent a link between the adaptive and the innate immune system. Additionally, NKTs release cytokines (TNF-α, IFN-γ, IL-17, IL-22) and chemokines (CCR5, CCR6, CXCR3), thus stimulating the infiltration of other immune cells [121,122,123]. In fact, NKs and NKTs may contribute to the induction and maintenance of the inflammatory state, as also observed in a murine model of psoriasis [122,123]. These cells recognize the antigen presented by the surface receptor CD1d [124] and are highly represented in psoriatic skin, suggesting their involvement in the pathogenesis of the disease [103].

### 2.5. Neutrophils

Neutrophils are the cells most involved in the innate immunity; in fact, these cells play an important defense role through a respiratory burst, resulting in the release of reactive oxygen radicals (ROS) and degranulation and formation of neutrophil extracellular traps (NETs) [125]. Their activity is also related to the interaction and communication with APC cells and lymphocytes [126,127]. In psoriatic plaque-injured skin, neutrophils work by increasing respiratory bursts and releasing proteases, such as neutrophil elastase (NE), cathepsin g, myeloperoxidase (MPO), and proteinase 3. These mechanisms contribute to the increase in oxidative stress processes with consequent employment of the chemical mediators of phlogosis and the formation of autoantigen, typical of the psoriatic lesion [128,129]. However, the abundant presence of neutrophils is considered a typical histopathological sign of psoriasis; in fact, the neutrophil/lymphocyte ratio (NLR) is significantly increased in psoriatic patients [130,131]. The role played by NETs in the pathogenesis of psoriasis appears crucial: increased levels have been observed in lesions and plaques of psoriatic skin. A consequence of this numerical overload is the augmented release of IL-17 due to a stronger cellular stimulation, with the further secretion of chemical mediators, which again leads to a self-amplification of the number of neutrophils [128].

### 2.6. Mast Cells

**Figure 3:**
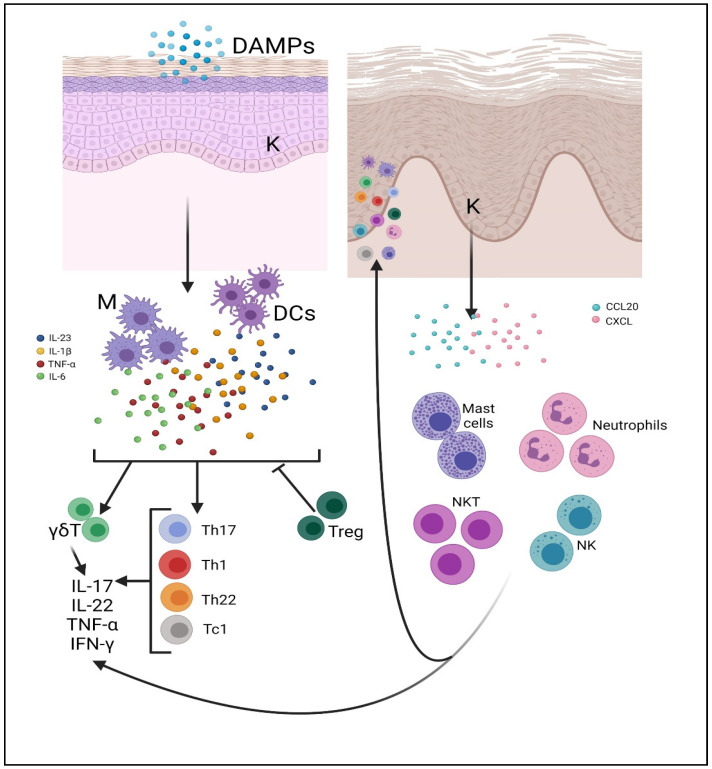
The pathogenetic mechanism of psoriasis. DAMPs activate DCs and macrophages by inducing the secretion of IL-23, IL-1β, IL-6, and TNF-α. They stimulate the TCD4+ (Th1, Th17, Th22) and CD8+ (Tc1)-mediated immune response. Mast cells, neutrophils, NK and NKT cells, and other immune cells infiltrate the site of phlogosis in the skin, contributing to disease progression by releasing IL-17 and antimicrobial peptides. Treg cells lose their suppressive activity and, in some cases, convert to Th17 cells, further increasing the local inflammatory reaction. Cytokines and chemokines act on keratinocytes, inducing their hyperproliferation. Activated keratinocytes produce CCL20 and CXCL1, 3, 8, 11, increasing cell infiltration. This creates an amplified continuous cycle that leads to injury. Abbreviations: M, macrophages; K, keratinocytes. Created with BioRender.com (accessed on 18 February 2022).

Mast cells are related to innate immunity and primarily responsible for acute inflammatory processes [132]. Once activated, these cells secrete several substances contained in the cytoplasmic granules such as heparin and histamine, nitric oxide (NO), leukotrienes, and interleukins such as IL-8, IL-17, and IL-22 [133,134]. Moreover, mast cells are active in skin lesions, contributing to the formation of psoriatic plaque [134]. The role of mast cells in psoriasis is mainly related to IL-22 production, whereas their role in IL-17 secretion is still unclear. IL-17 appears to be produced mainly by T cells and only to a small extent by mast cells [134], although other studies support the hypothesis that mast cells are involved in IL-17 production in both healthy and psoriatic skin [62]. The pathogenetic mechanism of immune cells in psoriasis in summarized in Figure 3.

## 3. Psoriasis-Related Psychological Alterations

A peculiar psychological and personality profile can be outlined in patients with psoriasis. Some studies have shown that patients with this disease generally have a type D (D for “distressed”) personality, with high levels of social inhibition (SI) and negative affectivity (NA) [135]. This means that these patients experience strong negative emotions, but they avoid expressing them because of their fear of being disapproved [136]. In addition, they are more likely to manifest early maladaptive patterns, including emotional deprivation and vulnerability to harm [137], which are significant predictors of psychological distress, leading to difficulties in communicating their needs and emotions [138]. Several studies show that the quality of life of these patients is much lower than that of healthy patients, and that its impact is similar to that of other major chronic diseases [139]. Psoriasis and mental disorders could also be considered in a syndemic context since they influence each other on both a physiological and social level, which may suggest that they may become acute or alleviate at the same time [140]. Therefore, according to this perspective, a psychological intervention could be effective in alleviating both psoriasis and mental disorders [141]. Cognitive behavioral psychotherapy (CBT) is effective in treating psoriasis, especially in patients with moderate or severe forms, in terms of both area of reduction and severity of the disease [142], as well as being useful in treating feelings of hopelessness and other related comorbidities [142].

### 3.1. Psoriasis and Depression

**Figure 4:**
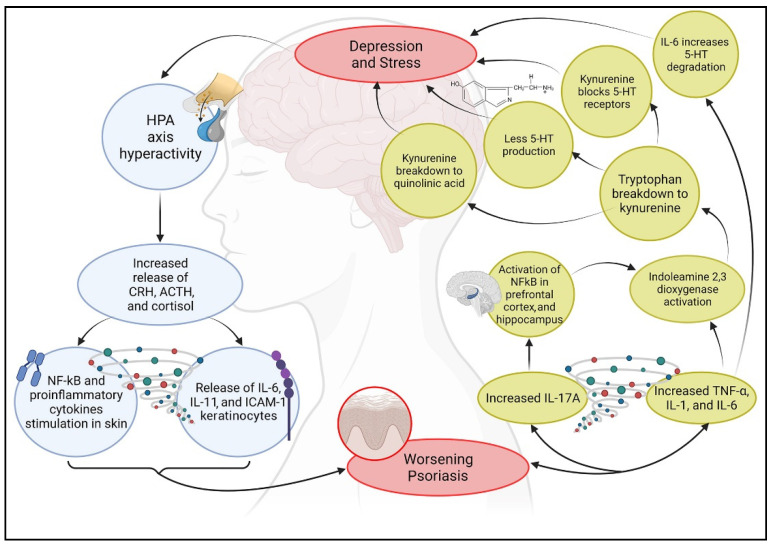
Interactive relationship between psoriasis, depression, and stress. This graphic scheme summarizes the biological cyclic mechanism discussed in this review. Created with BioRender.com (accessed on 14 March 2022).

Several studies have demonstrated a correlation between psoriasis and depression [143]. Kurd et al. (2010) examined the clinical reports of almost 1 million subjects, aimed to determine the incidence of levels of anxiety, suicide risk, and depression in both patients with psoriasis and the rest of the population [144]. The results suggest that psoriatic patients have a higher risk of developing depression [145] and anxiety and are at a higher risk of committing suicide, especially young and male patients, as well as having greater comorbidities with other psychiatric disorders [144]. Furthermore, several studies suggest that depressed patients show a 30% higher production of proinflammatory cytokines, such as IL-1β, TNF-α, and C-reactive protein (CRP) [146,147], which affect the metabolism of some neurotransmitters (such as dopamine, serotonin, and glutamate), neuroendocrine function, and even neuroplasticity, resulting in neurotoxicity and neuronal apoptosis [148,149]. This production of cytokines can play an important role in both the aggravation of psoriasis and depression, confirming the link between the immune and neuroendocrine system and human behavior, which is altered in these cases [146,150,151]. A mechanism that elicits the onset of depression and at the same time chronic inflammation in patients with psoriasis is the hyperactivity of the HPA axis, resulting in the release of increased levels of CRH, ACTH, and cortisol and in a stimulation of the transcription factor NF-kB and of the proinflammatory cytokines in the skin [152]. CRH stimulates the proinflammatory cytokines IL-6 and IL-11 as well as the expression of the intracellular adhesion molecule-1 (ICAM-1) of keratinocytes, which promotes the migration of immune cells and facilitates cell-mediated immune responses [153]. In addition, dysfunction of cortisol receptors (mineralocorticoids and glucocorticoids) downstream from the HPA axis reduces the sensitivity of the anti-inflammatory effects of cortisol since the body is unable to skillfully regulate corticosteroid levels (Figure 4) [154]. Recently, the role of Th17 cells in depression has been investigated [155]. Several studies found that these cells were augmented in the blood of depressed patients [156]. Patients with psoriasis who show high levels of cytokines, Th17 cells and IL-17A, are more subjected to develop depression and anxiety disorders [144]. These data suggest a strong correlation between depression and the immune system; therefore, a relationship between depression and psoriasis may be hypothesized [157]: depression improvement might be observed following treatment of psoriasis, and conversely, depression treatment might improve psoriasis symptoms [157].

### 3.2. Psoriasis and Stress

Stress influences the activity of the immune response by increasing the cascade of proinflammatory cytokines [158]. Two neurotransmitters are generally associated with stress and psoriasis: serotonin (5-HT) and dopamine (DOPA). Serotonin is a potent neurotransmitter involved in nervous, immune, and endocrine systems [159,160,161,162,163,164]. Low levels of 5-HT increase the production of some inflammatory mediators, such as TNF-α and IL-1β, which induces the activation and deterioration of keratinocytes via NF-kB, which is activated by IL-17A in the prefrontal cortex and in the hippocampus, thus worsening the symptoms of psoriasis [165,166]. Inflammation can also activate indoleamine 2,3-dioxygenase, which induces tryptophan, a precursor of 5-HT, to break down into kynurenine [167], an antagonist of serotonin receptors, thus inducing depressive symptoms despite 5-HT availability. Moreover, kynurenine can break down into quinolinic acid, a neurotoxin that accumulates in the anterior cingulate gyrus of depressed patients [168]. This whole process is also adjuvated by IL-6, which increases the degradation of serotonin in the brain [169]. The psoriatic inflammatory process can simultaneously decrease the production of serotonin and may inhibit 5-HT receptors, thus contributing to its reduction (Figure 4).

DOPA can regulate mast cell degranulation and consequently stimulates the release of proinflammatory cytokines [170]; therefore, dopamine might be considered a risk factor for psoriasis [171]. Both innate and adaptive immune systems are involved in the stress response. Similar to what happens in the skin, damage-associated molecular patterns stimulate innate immune cells [172] to produce different interleukins and TNF-α [173] and to recruit an increasing number of monocytes that begin to circulate in the blood [174]. This unresolved inflammatory state can lead to chronic psychological stress [175]. The adaptive immune system can activate T cells in response to stress, thus stimulating cytokine release and NK cell and DC recruitment [176]. Consequent to stress, the sympathetic nervous system can endorse amine secretion, thus provoking the proliferation of myeloid cells (such as monocytes). These pieces of evidence suggest a link between stress and etiology of psoriasis.

## 4. Natural Compounds as an Alternative Treatment for Psoriasis

**Figure 5:**
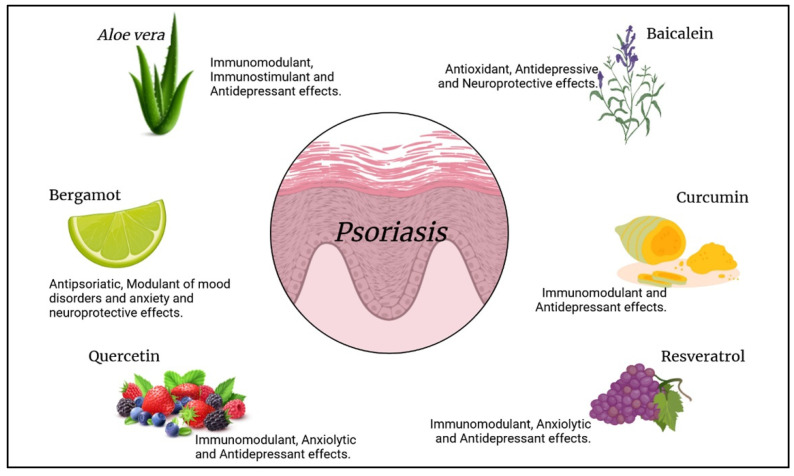
Natural compounds and their effects on psoriasis. Created with BioRender.com (accessed on 14 March 2022).

No definitive treatment is currently available to treat psoriasis; however, some biological therapies have shown encouraging results [177,178,179]. In particular, treatment with monoclonal antibodies, such as infliximab, ixekizumab, risankizumab, bimekizumab, guselkumab, secukinumab, and brodalumab, determined excellent outcomes in patients with moderate to severe psoriasis [180]. These drugs are mainly anti-TNF-α, anti-IL12/23, anti-IL-17, and anti-IL-23 antibodies and demonstrate the significant involvement of these patterns in exacerbating psoriasis and the therapeutic significance of using them as targets [180]. However, the long-term administration of these drug is often related to the appearance of different side effects [180,181]; for this reason, other therapeutic approaches and natural medicine have recently gained much attention from the scientific world. Natural products are potentially rich in bioactive compounds; in fact, different studies have already shown that some of these natural extracts may play anti-inflammatory, antioxidant, and antiproliferative effects [182,183]. These pieces of evidence demonstrate their possible use for the treatment of different diseases and also of psoriasis even thanks to their safety and the possible better compliance of patients [184,185,186]. In the following section, some of the best-known natural compounds with antipsoriatic properties are reported (Figure 5).

### 4.1. Aloe vera L.

*Aloe vera* L., a plant belonging to the *Liliaceae* family, contains polysaccharides, salicylic acid, and vitamins that have anti-inflammatory and antipruritic properties [187]. *Aloe vera* possesses an immunomodulating effect, stimulating macrophages and lymphocytes to release NO and cytokines and activating the maturation of undeveloped dendritic cells [187]. A study conducted by Leng et al. (2018) showed that *Aloe vera* can inhibit overexpression of keratinocytes and overregulation of the NF-κB signaling pathway [188]. Clinical and preclinical studies demonstrated that ethanolic extract of *Aloe vera* exerts a positive activity on psoriatic lesions, similar to traditional drugs [189]. In addition, *Aloe vera* may have a significant antidepressant effect. The intake of at least 500 mg of *Aloe vera* capsules reduced depressive state in patients after 8 weeks of treatment [190]. The concomitant use of fluoxetine, a selective serotonin reuptake inhibitor (SSRI), reduces depressant symptoms in a mouse model [191].

### 4.2. Bergamot Essential Oil

Bergamot is the common name for *Citrus bergamia*, a plant belonging to the *Rutaceae* family [192]. Bergamot essential oil is one of the herbal preparations derived from *C. bergamia*, and it exerts antibacterial properties [193] and can help in the moderation of mood disorders and stress-induced anxiety, as well as sleep induction, and neuroprotection [194]. A study by Valkova et al. (2007) showed that the combination of bergamot essential oil and UVB rays significantly reduced the symptoms of psoriatic lesion [195]. This is probably due to the action of 5-methoxypsoralen (5-MOP), the major constituent of bergamot essential oil, mainly used in cosmetics and medicine. The application of this molecule in herbal medicine is useful, in fact, to combat skin damage, such as psoriasis-induced skin lesions and vitiligo [196]. Moreover, bergamot essential oil has been shown to be useful in neuroprotection (even during experimental brain ischemia), chronic pain control, and the management of stress, anxiety, and anxiety-related conditions in neuropharmacological research [197].

### 4.3. Quercetin

Quercetin, well known for its antioxidant and anti-inflammatory activity [198], can induce apoptosis through ROS generation [199] and can support keratinocyte-growth inhibition induced by arsenic trioxide [200]. A study by Mestry et al. (2020) evaluated the beneficial effect of a gel based on quercetin combined with *Commiphora mukul* in the treatment of psoriasis [201]. Moreover, on the one hand, quercetin decreased TNF-α, IL-6, and IL-17 levels, modulating the inflammatory state in psoriasis [202], and on the other hand, it is also involved in the management of cholinergic and serotonergic functions, producing an anxiolytic and antidepressant effect and enhancing memory performance [203]. A recent study has confirmed that quercetin significantly reduces anxiety behaviors in mice subjected to mild traumatic brain injury and regulates ACTH and corticosterone levels in the HPA axis, enhancing depressive states [204].

### 4.4. Baicalein

Baicalein, a traditional Chinese drug with anti-inflammatory and antiviral effects [205], can regulate keratinocyte proliferation and differentiation [206]. It can decrease the severity of chronic inflammations by improving the antioxidant status and reducing the oxidative stress, regulating the secretion of cytokines and chemokines (IL-6 and TNF-α reduction) [207,208], inhibiting Th17 activation, and blocking the IL-17-induced inflammatory cascade. Furthermore, baicalein can improve depressive symptoms, preventing the loss of dopamine and brain-derived neurotrophic factor (BDNF) [183,209]. In particular, a study on a murine model highlighted that baicalein treatment can alleviate depression-like symptoms [210].

### 4.5. Curcumin

Curcumin can be used for the treatment of psoriasis. In fact, the active constituent of *Curcuma longa* [211] shows an antiproliferative effect on keratinocytes [181]. Moreover, curcumin may have significant anti-inflammatory effects by decreasing cytokine levels, such as IL-1β, IL-6, IL-22, and TNF-α, suggesting new perspectives for its therapeutic use [212]. Furthermore, curcumin inhibits phosphorylase kinases, which are increased in patients with psoriasis [213]. Curcumin also showed a strong antidepressant effect [214] thanks to its modulation of neurotransmitters, such as noradrenaline, dopamine, serotonin, and monoamine, and through moderation of excessive corticosterone secretion, which causes dysfunction in the HPA axis [215].

### 4.6. Resveratrol

Resveratrol is a polyphenol with anti-inflammatory and antioxidant properties contained in several foods, especially red wine, which is a key element in the Mediterranean food tradition [216,217,218,219]. Resveratrol acts by reducing the secretion of inflammatory cytokines and inducing apoptosis in keratinocytes via silent mating type information regulation 2 homolog (SIRT1). Resveratrol may act by inhibiting the release of IL-17 [220]. In addition, a study by Khurana et al. (2020) proved that resveratrol-loaded polymeric micelles act on psoriatic lesions with positive dermatological results [221]. A recent study showed that resveratrol also plays a critical role in neuroprotection, inhibiting the expression of phosphodiesterase 4D (PDE4D), an enzyme that catalyzes the hydrolysis of cyclic adenosine monophosphate (cAMP). PDE4D regulates cAMP expression at the intracellular level, reducing the depressant- and anxious-like states of corticosterone induced by cell lesion on a mouse model [222].

In summary, natural products analyzed here show antipsoriatic, antidepressant, and anxiolytic activities.

The biological effects of natural compounds are summarized in Table 1.

## 5. Conclusions

Psoriasis is the most common chronic autoimmune skin disease that seems to arise from the interaction between external and internal factors, as well as vitiligo. High stressogenic conditions and alterations in the psychological picture seem to play a key role in the development of these diseases. It results from a chronic inflammatory state orchestrated by cells of the immune system, such as lymphocytes, mast cells, dendritic cells, and NK cells, which interact with keratinocytes. Since not all pathogenetic mechanisms related to these cells have been clarified, this review aims to evaluate the state of the art on the etiology of psoriasis and to offer further reflection points. In addition, no definitive treatment for psoriasis is available until now: commercial drugs cannot be administered for long times, so the use of alternative treatments can improve the clinical, physical, and psychological features of patients suffering from this disease. Further clinical and preclinical studies should be conducted to adopt natural compound therapy as an adjunct to traditional medicines, as these compounds act on the immune system, the skin, and the central nervous system. In conclusion, since the inflammatory psoriatic state results from uncontrolled activation of immune system cells, further studies on natural bioactive compounds that inhibit or suppress these hyperactivations could be suggested.

## Figures and Tables

**Table 1:** Biological effects of natural compounds on psoriasis.

Natural Compounds	Biological Effects	References
*Aloe vera* L.	Immunomodulant and stimulant effect on macrophages, lymphocytes, and dendritic cells.	[187]
	Antidepressant effect.	[190,191]
Bergamot essential oil	Antibacterial and anti-psoriatic properties.	[193,195]
	Moderation of mood disorders and stress-induced anxiety. Neuroprotective and sleep-inducing effect.	[194]
Quercetin	Decrease in TNF-α, IL-6, and IL-17 levels.	[202]
	Regulation of cholinergic and sero-tonergic functions, anxiolytic and anti-depressant effects, enhances memory performance.	[203,204]
Baicalein	Antioxidant effect, inhibits the release of IL-17 and the expression of IL-6 and TNF-α.	[207,208]
	Antidepressive and neuroprotective effect.	[183,209]
Curcumin	Decrease in IL-1β, IL-6, IL-22, and TNF-α levels.	[212]
	Modulates serotonin, monoamine, noradrenaline, and dopamine. Regu-lates the function of the HPA axis.	[214,215]
Resveratrol	Reduces the secretion of inflammatory cytokines and induces apoptosis in keratinocytes. Inhibits the release of IL-17.	[220]
	Anxiolytic and antidepressant effect by inhibiting the expression of PDE4D, which regulates cAMP expression at the intracellular level.	[222]

## Data Availability

Not applicable.

## Sample Availability

Not available.
